# Endometriosis does not seem to be an influencing factor of hypertensive disorders of pregnancy in IVF / ICSI cycles

**DOI:** 10.1186/s12958-022-00922-5

**Published:** 2022-03-25

**Authors:** Pingyin Lee, Canquan Zhou, Yubin Li

**Affiliations:** 1 Reproductive Medicine Center, The First Affiliated Hospital of Sun Yat-sen University, Zhoushan 2 Road, Guangzhou, Guangdong People’s Republic of China; 2 Guangdong Provincial Key Laboratory of Reproductive Medicine, The First Affiliated Hospital of Sun Yat-sen University, Zhoushan 2 Road, Guangzhou, Guangdong People’s Republic of China

**Keywords:** Endometriosis, Hypertensive disorders of pregnancy, In vitro fertilization, Frozen-thawed embryo transfer

## Abstract

**Introduction:**

To evaluate whether the incidence of hypertensive disorders of pregnancy (HDP) in pregnant women was related to endometriosis (EM), ovulation and embryo vitrification technology.

**Methods:**

A retrospective cohort study was conducted on the clinical data of 3674 women who were treated with IVF / ICSI in the Reproductive Medicine Center of the First Affiliated Hospital of Sun Yat-sen University and maintained clinical pregnancy for more than 20 weeks. All pregnancies were followed up until the end of pregnancy. The follow-up consisted of recording the course of pregnancy, pregnancy complications, and basic situation of newborns.

**Results:**

Compared with NC-FET without EM, HRT-FET without EM was found to have a higher incidence of HDP during pregnancy (2.7% V.S. 6.1%, *P*<0.001); however, no significant difference was found in the incidence of HDP between NC-FET and HRT-FET combined with EM (4.0% V.S. 5.7%, *P*>0.05). In total frozen-thawed embryo transfer (total-FET), the incidence of HDP in the HRT cycle without ovulation (HRT-FET) was observed to be higher than that in the NC cycle with ovulation (NC-FET) (2.8% V.S. 6.1%, *P*<0.001). In patients with EM, no significant difference was found in the incidence of HDP between fresh ET and NC-FET (1.2% V.S. 4.0%, *P*>0.05).

**Conclusion:**

EM does not seem to have an effect on the occurrence of HDP in assisted reproductive technology. During the FET cycle, the formation of the corpus luteum may play a protective role in the occurrence and development of HDP. Potential damage to the embryo caused by cryopreservation seems to have no effect on the occurrence of HDP.

## Background

Endometriosis (EM) refers to the presence of functional endometrial tissue (glands and stroma) outside the uterus, in which its incidence in women of childbearing age is 5-10% [1, 2]. EM is characterized by estrogen dependent chronic inflammation, which often manifests as dysmenorrhea, lower abdominal pain, dyspareunia and infertility [3].

Many studies have shown that EM can increase the risk of multiple adverse pregnancy outcomes, such as spontaneous abortion, ectopic pregnancy, hypertensive disorders of pregnancy (preeclampsia or gestational hypertension), gestational diabetes mellitus (GDM), preterm birth and low birth weight [4–6]. In addition, the immune system and inflammation have been considered to serve as pivotal factors in disease progression [7]. Abnormal hormone circulation from EM as well as pathological changes due to chronic inflammation may lead to a higher risk of hypertension [8]. At present, the pathogenesis of hypertensive disorders of pregnancy (HDP) has yet to be fully elucidated. In pregnancy, different placental conditions might damage the maternal endothelium, making this dysfunction the common gateway for HDP [9]. HDP are a heterogeneous group of conditions that include chronic hypertension, gestational hypertension, preeclampsia eclampsia, and chronic hypertension with superimposed preeclampsia [10]. It is generally believed that the occurrence of HDP may be related to immune system disorder, trophoblast or placental ischemia and oxidative stress [11, 12]. Studies have also shown that the incidence of HDP is higher when using assisted reproductive technology (ART), especially in the frozen-thawed embryo transfer (FET) cycle [13], which may be related to the endometrial preparation protocol in the FET cycle [14].

Bellac et al [15] and Lani et al [16] have shown that EM can significantly increase the risk of HDP. However, Hadfield et al [17] found that EM did not increase the risk of HDP, with certain studies showing that EM could reduce the risk of HDP [18]. Different opinions exist regarding EM and HDP, though none are conclusive, and studies pertaining to ART are scarce. Furthermore, both EM and HDP are known to be involved in abnormal immune factors, and immune factors are extremely complex. Accordingly, whether EM has an influence on the occurrence of HDP is worthy of further discussion.

Therefore, by analyzing women of more than 20 weeks of clinical pregnancy who were treated with IVF / ICSI (intracytoplasmic sperm injection, ICSI) at our reproductive center in the past 4 years, this study aims to determine whether the risk of HDP is related to EM, ovulation and embryo vitrification technology.

## Materials and methods

### Study design and setting

This study was a retrospective cohort study that analyzed the clinical data of 3674 patients treated with IVF / ICSI at the Reproductive Medicine Center of the First Affiliated Hospital of Sun Yat-sen University from January 2017 to June 2021. The protocol was reviewed and approved by the Ethical Committee of The First Affiliated Hospital of Sun Yat-Sen University. The patients/participants provided their written informed consent to participate in this study.

**Fig. 1:**
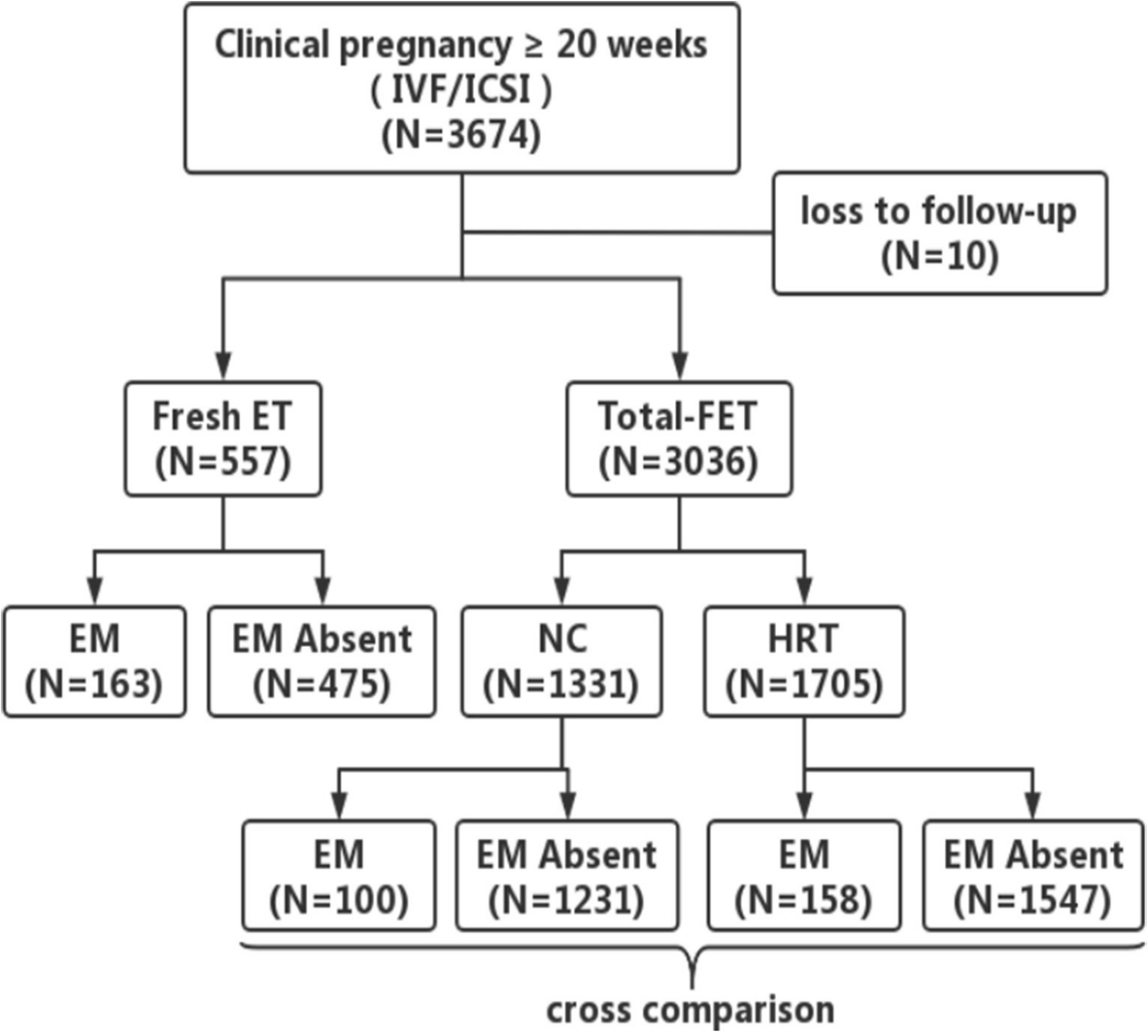
A flow diagram showing the distribution of the study populations

The study included 638 cycles of fresh embryo transfer (fresh ET) and 3036 cycles of FET. In order to distinguish FET from HRT-FET and NC-FET, total-FET was used to represent the overall FET cycle (Fig. 1). The inclusion criteria of the EM group were as follows: patients aged 20-45 years; diagnosed with endometriosis; has intrauterine gestational sac pregnancy under B-ultrasound 1 month after embryo transfer; and clinical pregnancy status has been maintained for more than 20 weeks. The inclusion criteria of the EM absent group were as follows: patients aged 20-45 years and undergoing IVF / ICSI treatment due to tubal and/or male factors infertility. Exclusion criteria included: diagnosed with PCOS, hyperprolactinemia, abnormal thyroid function, and chromosome abnormalities of one or both of other endocrine related diseases that are not conducive to pregnancy or can cause adverse pregnancy; has underwent ovarian, thyroid, pituitary surgery or antitumor radiotherapy and chemotherapy; undergone oophorectomy due to ovarian malignancy or other reasons.

Women were asked whether they had physician diagnosed EM. Participants who responded “yes” indicated the year of diagnosis and whether it had been visually confirmed by laparoscopy, the clinical gold standard for endometriosis diagnosis [19]. For participants who answered "yes" but did not diagnose EM by laparoscopy can still be classified as EM only after being reported as EM by B-ultrasound. The remaining patients who were not diagnosed with EM by laparoscopy and those who were reported as EM by B-ultrasound during the treatment were also classified as EM. This inclusion is based on the comprehensive consideration of the patient's reproductive age and needs. We considered women to have a HDP in a given pregnancy if they had a diagnosis of gestational hypertension, pre-eclampsia, eclampsia, chronic hypertension with superimposed preeclampsia or HELLP syndrome on the medical record at any time between one month before delivery and seven days post partum [20]. Women who could not be included in HDP: who had a history of asthma complications, known coronary artery disease, type 1 diabetes with microvascular complications, signs of heart failure, or clinical dissection of the aorta were ineligible [21]. To be considered exposed to a HDP in our study, women with diagnoses registered outside this time window also had to have at least one diagnosis registered within the window. (We adopted this restriction to try to ensure that diagnoses of hypertensive disorders of pregnancy reflected true cases.)

### Stimulation protocol

According to the patient's age, body mass index (BMI), basic sex hormone level, anti-Mullerian hormone (AMH) and antral follicle count (AFC), the appropriate stimulation protocol and starting dose were selected. Ovulation was triggered using 250 μg of recombinant hCG (Ovidrel, Merck-Serono, Switzerland) or 5000-10000 IU hCG (Lizhu, Zhuhai, China) when two follicles reached 18 mm or three follicles reached 17mm in diameter. Transvaginal ultrasound-guided oocyte retrieval was performed 34-36 hours later. Following oocyte retrieval, whether to carry out fresh ET or whole embryo freezing was determined according to whether the patient had high ovarian hyperstimulation syndrome (OHSS) risk, high progesterone level, uterine cavity and condition of endometrium. If fresh ET was planned, Progesterone Sustained-release vaginal gel (Crinone, Merck-Serono, Switzerland) 90mg/d or intramuscular progesterone 40mg/d was given on the day of oocyte retrieval. Dydrogesterone (DydrogesteroneTablets, Abbott biologicals, Netherlands) 10mg was orally administered twice a day until 14 days following embryo transfers.

### Fertilization and embryo culture

After culturing 2-6h in vitro, IVF or ICSI was selected according to the condition of the male semen. The number and size of prokaryotes, number and distribution of nucleolar precursor bodies and cytoplasmic distribution were observed 16-18 hours after fertilization. Meanwhile, following 72 h, D3 cleavage embryos were scored according to the number of blastomeres, uniformity of blastomeres size, amount and distribution of fragments. Embryo morphology was evaluated according to the Istanbul Consensus Workshop on Embryo Assessment [22].

### Embryo transfer

No more than two cleavage stage embryos were transferred on the morning of the 3rd day after oocyte retrieval. According to our embryo culture strategy, if there were no more than two cleavage stage embryos available after fresh embryo transfer, they were vitrified on the 3rd day. Otherwise, the remainder had blastocyst culture performed. Surplus cleavage stage embryos or blastocysts were vitrified using the Cryotop (Kitazato Supply Co.,Fujinomiya, Japan) method [23] for subsequent FET cycles whenever necessary. Embryo morphology was evaluated according to the Istanbul Consensus Workshop on Embryo Assessment [22].

During the FET cycles, endometrial preparation protocols included hormone therapy (HRT) cycles and natural cycles (NC), as previously described in detail [24]. All embryo transfers were performed under transabdominal ultrasound guidance.

### Determination of clinical pregnancy and follow-up

Serum hCG levels were determined 12-14 days after embryo transfers. A clinical pregnancy was confirmed by transvaginal ultrasound 3 weeks after a positive serum HCG test. Luteal support was continued to the 10th week of gestation. All pregnancies were followed up by our staff until the end of gestation. The details relevant to the follow-up were recorded, including the course of pregnancy, delivery time, mode of delivery, complications during pregnancy, gender, birth weight and congenital abnormalities of newborns.

### Statistical analysis

According to the calculation results of G. Power software, in the two main research indicators (EM and HDP) of this study, when the sample size is N = 450, it can be ɑ= 0.05 provides more than 80% statistical power. If the loss of follow-up rate is 20%, the total sample size to be included in this study shall be at least N = 540. A total of 3674 patients were recruited in this study, and the sample size was sufficient.

Categorical data were presented as numbers and percentages. Continuous variables were given as mean±SD. The chi-square test or Fisher exact probability test was used for categorical variables, while ANOVA was done for continuous variables. Bonferroni method was utilized for pairwise comparison between each group. A p-value < 0.05 was considered to be statistically significant.

## Results

### Baseline characteristics and clinical data of patients

A total of 3674 patients were included, which included 638 cycles of fresh ET and 3036 cycles of total-FET. No significant difference was observed between the two groups in regard to age, infertility years, female BMI, endometrial thickness during ET, gender of live birth (single fetus), number of losses to follow-up, incidence of HDP and EM (*P*>0.05). There were significant differences between the two groups in the number of embryos transferred, number of gestational sacs and number of live births (*P*<0.05) (Table 1). Among the 3036 cycles of total-FET, there were 1331 cases of natural cycles (NC-FET) and 1705 cases of hormone therapy cycles (HRT-FET). Moreover, no significant difference was noted between the two groups in terms of age, infertility years, female BMI, endometrial thickness during FET, number of embryos transferred, number of gestational sacs, number of live births, gender of live birth (single fetus), number of losses to follow-up, incidence of HDP and EM (*P*>0.05) (Table 2).

**Table 1:** Baseline characteristics and clinical data of all included patients

Characteristic	Fresh ET cycles	Total-FET cycles	p-value
N	638	3036	
Female age (y)	32.30 ± 4.13	32.18 ± 4.39	0.556
Male age (y)	34.81 ± 5.77	34.64 ± 5.36	0.325
History of infertility (y)	3.56 ± 2.76	3.77 ± 2.29	0.785
Female BMI (kg/m2)	21.36 ± 2.68	21.27 ± 3.13	0.946
Endometrial thickness during ET	10.93 ± 2.11	9.29 ± 1.36	0.537
Number of embryos transferred	1.89 ± 0.30	1.21 ± 0.40	0.039
Number of gestational sac by ultrasound			<0.001
1	439	2425	
2	196	597	
3	3	13	
4	0	1	
Number of live births			<0.001
1	426	2542	
2	120	422	
3	0	4	
During pregnancy	80	0	
Middle and late stage abortion or induced labor / Stillbirth	11	59	
Gender of live birth (single fetus)			0.682
Male	224	1367	
Female	202	1175	
loss to follow-up	1	9	0.603
Endometriosis			0.069
Present	163	258	
Absent	475	2778	
Hypertensive disorders of pregnancy			
Present	19 (11.9%)	141 (17.6%)	0.061
Absent	619 (88.1%)	2895 (82.4%)	

Note: Data are presented as mean (standard deviation) or N, unless stated otherwise

**Table 2:** Baseline characteristics and clinical data of patients in total-FET cycles

Characteristic	NC-FET	HRT-FET	p-value
N	1331	1705	
Female age (y)	32.18 ± 4.36	32.18 ± 4.42	0.576
Male age (y)	34.44 ± 5.28	34.80 ± 5.42	0.235
History of infertility (y)	3.46 ± 2.28	3.47 ± 2.49	0.675
Female BMI (kg/m2)	21.37 ± 2.13	21.30 ± 3.06	0.876
Endometrial thickness during FET	9.34 ± 0.65	9.27 ± 0.23	0.834
Number of embryos transferred	1.17 ± 0.37	1.20 ± 0.40	0.123
Number of gestational sac by ultrasound			0.593
1	1090	1335	
2	233	364	
3	7	6	
4	1	0	
Number of live births			0.113
1	1126	1416	
2	183	239	
3	2	2	
During pregnancy	0	0	
Middle and late stage abortion or induced labor / Stillbirth	19	40	
Gender of live birth (single fetus)			0.447
Male	596	771	
Female	530	645	
loss to follow-up	1	8	0.113
Endometriosis			0.086
Present	100	158	
Absent	1231	1547	
Hypertensive disorders of pregnancy			
Present	37 (2.8%)	104 (6.1%)	<0.001
Absent	1294 (97.2%)	1601 (93.9%)	

Note: Data are presented as mean (standard deviation), or N and incidence (%), unless stated otherwise

### Relationship between EM, HDP and endometrial preparation protocol during all included cycles

In the total-FET cycle, the incidence of HDP in the HRT cycle was found to be higher than that in the NC cycle (*P*<0.05) (Table 2). After controlling the effects of number of gestational sacs by ultrasound and live births, fresh ET and total-FET were analyzed, in which no significant difference was found in the incidence of HDP during pregnancy in the fresh ET cycle and total-FET cycle, regardless of whether EM was combined (*P*>0.05) (Table 3). The two types of endometrial preparation protocols were further compared and were analyzed as to whether they had EM. Accordingly, in the total-FET cycle, the incidence of HDP during pregnancy was noted to be higher in the HRT cycle without EM than in the NC cycle without EM (6.1% V. S 2.7%) (*P*<0.05). Meanwhile, no significant difference was found in the incidence of HDP between the NC and HRT cycles with EM (*P*>0.05) (Table 4).

**Table 3:** Relationship between EM and HDP in all included cycles

			Hypertensive disorders of pregnancy		Significance (2-sided)
			Present	Absent	
Fresh ET	EM	Present	2 (1.4%)	143 (98.6%)	0.181
		Absent	17 (4.1%)	395 (95.9%)	
	Total		19	538	
Total-FET	EM	Present	13 (5.0%)	245 (95.0%)	0.763
		Absent	128 (4.6%)	2650 (95.4%)	
	Total		141	2895	

Note: Data are presented as N and incidence (%), unless stated otherwise

**Table 4:** Relationship between EM, endometrial preparation protocol and HDP in total-FET cycle

	Hypertensive disorders of pregnancy		Significance (2-sided)
	Present	Absent	
NC	33 (2.7%) b	1198 (97.3%) b	<0.001
NC+EM	4 (4.0%) a,b	96 (96.0%) a,b	
HRT	95 (6.1%) a	1452 (93.9%) a	
HRT+EM	9 (5.7%) a,b	149 (94.3%) a,b	
Total	141	2895	

Note: Data are presented as N and incidence (%), unless stated otherwise. The letters (a, b) in each footmark are the results of the comparison between the two groups. If the same letters were present, it means that there was no statistical significance between them (*P* > 0.05)

### Relationship between ovulation cycle (fresh ET cycle, NC-FET cycle) and HDP in EM

In all cases of ovulation (fresh ET cycle, NC-FET cycle), no significant difference was found in the incidence of HDP during pregnancy between fresh ET cycles with EM and NC-FET cycles with EM (*P*>0.05) (Table 5).

**Table 5:** Relationship between fresh ET cycle, NC-FET cycle and HDP in EM

	Hypertensive disorders of pregnancy		Significance (2-sided)(Fisher)
	Present	Absent	
Fresh ET + EM	2 (1.2%)	161 (98.8%)	0.205
NC + EM	4 (4.0%)	96 (96.0%)	
Total	6	257	

Note: Data are presented as N and incidence (%), unless stated otherwise

### Relationship between EM, embryo transfer methods and HDP in singleton pregnancies

According to the previous retrospective analysis of the total population, a few twin pregnancies were present in each group, which may interfere with the results. In order to be more thorough, an analysis of singleton pregnancy was carried out separately. Here, no significant difference was found between the incidence of HDP and whether EM was combined (OR 0.881, 95% CI 0.468-1.658, *p*>0.05), different embryo transfer methods (fresh ET, total-FET) (OR 0.617, 95% CI 0.328-1.158, *p*>0.05) or the gender of live birth (OR 1.107, 95% CI 0.761-1.608, *p*>0.05). In different ovulation cycles of total-FET (NC, HRT), it can be found that the incidence of HDP in NC is lower (OR 0.421, 95% CI 0.270-0.658, *p*<0.05) (Tables 6 and 7).

**Table 6:** Relationship between EM, embryo transfer methods, gender of live birth and HDP in singleton pregnancies

		Hypertensive disorders of pregnancy		Significance (2-sided)
		Present	Absent	
EM	Present	11 (3.5%)	303 (96.5%)	0.877
	Absent	105 (4.0%)	2548 (96.0%)	
ET	Fresh ET	11 (2.6%)	414 (97.4%)	0.138
	Total-FET	105 (4.1%)	2437 (95.9%)	
Total-FET	NC	27 (2.4%)	1099 (97.6%)	<0.001
	HRT	78 (5.5%)	1338 (94.5%)	
Gender of live birth	Male	65 (4.1%)	1526 (95.9%)	0.635
	Female	51 (3.7%)	1325 (96.3%)	
Total		116	2851	

Note: Data are presented as N and incidence (%), unless stated otherwise

**Table 7:** Cross analysis showing the effect on HDP of EM, embryo transfer methods, ovulation cycle and gender of live birth

	Odds Ratio	[95% CI]	
EM	0.881	0.468	1.658
ET (Fresh ET vs Total-FET)	0.617	0.328	1.158
Total-FET (NC vs HRT)	0.421	0.270	0.658
Gender of live birth (Male vs Female)	1.107	0.761	1.608

Note: Cross analysis was undertaken using Chi-square

## Discussion

EM is known to be common gynecological disease that causes infertility and may lead to adverse pregnancy outcomes, seriously placing the physical and mental health of women of childbearing age at risk along with safety of perinatal mothers and children. However, in recent years, the incidence of HDP in pregnant women with EM has remained controversial. The results of this study demonstrated that no difference in the incidence of HDP was observed in total-FET regardless of whether EM was combined (Table 3). After further cross comparison of the endometrial preparation protocol with EM, the incidence of HDP during pregnancy in HRT cycle without EM (6.1%) was found to be higher than that in the NC cycle without EM (2.7%), though no significant difference was present in the incidence of HDP between NC with EM (4.0%) and HRT with EM (5.7%) (Table 4). In the results of singleton pregnancies that were delivered alive, we also found that the incidence of HDP in HRT was higher (Tables 6 and 7). Notably, in the corresponding data, the incidence of HDP in all EM groups (4.0% and 5.7%) was within the HDP global incidence rate (5%-10%) [25].

In terms of the results, after adding EM, the HDP incidence that should have been different between NC and HRT exhibited no differences (Table 4), which may suggest that EM improves the incidence of HDP in NC or reduces the incidence in HRT. However, there may be no statistical difference as the present case data are small. Accordingly, in the future, the sample size should be expanded for further research. Nevertheless, the present results suggest that there may be confounding factors in the total-FET cycle. Therefore, it is suggested that when analyzing HDP incidence, it is more appropriate to separate the NC cycle with ovulation from the HRT cycle.

Most studies have not confirmed the correlation between EM and HDP. In 2012, Vercellini et al. followed up with pregnancies of patients who underwent EM surgery. Among them, there were 150 patients with deep infiltrating endometriosis (DIE) whose lesions involved the vaginal rectal septum, for which the incidence of HDP did not increase in these patients [26]. Another study on the pregnancy of DIE patients following laparoscopic ureterolysis found that the incidence of HDP was 3.8% [27], which was not higher than the global incidence of HDP of 5% - 10% [25]. However, Nirgianakis et al. put forward that the incidence of HDP in patients with pelvic DIE is higher than that in patients without EM [28]. Exacoustos et al. studied 52 patients with posterior pelvic DIE lesions ≥ 2 cm and found that the risk of HDP in patients with posterior pelvic DIE was increased, about 14.6%, which was significantly higher than that in patients without EM [29], though the baseline of the two groups of patients in terms of age, pregnancy and delivery times and BMI were not consistent, and the study did not exclude interfering factors, such as ART, for pregnancy. Some studies also found that the incidence of pre-eclampsia in EM patients has increased [30, 31]; however, these studies did not indicate whether patients assisted by ART were excluded, which cannot present a positive solution between EM and HDP. This study investigated the relationship between EM and HDP in IVF / ICSI. Currently, few articles exist on EM and HDP in ART, which gives the findings of this study more clinical value. Nevertheless, this study did not stratify the severity of EM and did not rule out that different degrees of EM may have different effects on the incidence of HDP, which will be investigated in the next step.

In the past decade, FET has significantly increased due to the expansion of surgical indications [32]. At present, numerous studies have shown that the risk of HDP associated with IVF has increased, especially in regard to FET [13, 33]. However, most reports have directly compared fresh ET and FET, where it was found that FET has a higher risk of HDP [34–36], though it does not clearly indicate which endometrial preparation protocol was used in FET. In this study, no difference was noted in regard to the incidence of HDP between fresh ET and total-FET (Table 1), in which the incidence of HDP in the HRT-FET cycle was found to be higher than that in the NC-FET cycle (Table 2). The results of this study are consistent with that of other research. In a big data retrospective analysis conducted in Japan, it was found that, compared with patients with natural ovulation cycle (NC-FET), patients using HRT-FET had an increased risk of HDP and placental implantation, while the risk of GDM was observed to be reduced [37]. Another retrospective cohort study in China also found that the HRT-FET group had an increased risk of HDP and placenta previa compared to ovarian stimulation in the FET group [14]. These results suggested that endometrial preparation methods may be related to obstetric complications, especially with respect to the development of HDP. This may be due to the increased risk of HDP from the lack of corpus luteum (CL) in patients with HRT-FET [38].

Cryopreserved embryos must be transferred to the uterus during the critical endometrial window that can establish pregnancy [39]. In reproductive women, the common endometrial preparation protocol of FET are NC, stimulated cycle and HRT cycle. In a natural cycle, the major follicle matures and produces E2, which leads to the development and thickening of the endometrium. Ovulation can then occur naturally, and the ovulation site becomes CL, which belongs to a functional ovarian cyst. In the stimulated cycle, ovulation was induced with either clomiphene citrate, letrozole, or gonadotropins, which may lead to one or more CL. However, in the HRT cycle, exogenous E2 and P lead to the development of the endometrium. During this period, the ovary is inhibited, hence, no dominant follicle, ovulation and CL exist. In contrast to the fresh cycle, there may be more CL in light of the role of the stimulation protocol.

The hypothesis that the HRT cycle without CL increases the risk of HDP seems to be biologically reasonable. CL can produce E2 and P as well as vasoactive products, such as relaxin, vascular endothelial growth (VEGF) and angiogenic metabolites of estrogen [40–42]. CL serves as an important source of reproductive hormones before the placenta becomes a source of reproductive hormones (such as P and E2) to maintain pregnancy. Vasoactive products produced by CL are very important for the formation of the initial placenta, and previous studies have proposed that the abnormal formation of early placenta is a critical step in the development of preeclampsia [43–45]. Since the HRT cycle does not form CL, relaxin and VEGF are not replaced when compared with other endometrial preparation protocols that involves CL formation, which is the pathological basis of subsequent pregnancy complications in women with an HRT cycle. Overall, these studies support the premise that CL deletion is associated with circulatory adaptation defects during pregnancy, in which the formation of CL may play a protective role in the occurrence and development of HDP. However, its mechanism remains unclear and requires further study.

In regard to the total-FET cycle, EM does not seem to affect the occurrence and development of HDP, whereas CL may influence them. In order to compare the effect of fresh ET and FET on HDP, EM patients in a fresh ET cycle were compared with EM patients in a total-FET cycle, where no difference in the incidence of HDP was found between the two groups (Table 5). These results suggested that FET does not cause maternal complications during pregnancy due to potential damage from the freeze-thaw process related embryo.

The number of FET cases has risen significantly within the past ten years, partly owing to improvements associated with vitrification compared with older slow-freeze methods [32]. However, no final conclusion was reached on whether vitrification technology can cause damage to embryos. Moreover, a cross-sectional analysis of 10744 transfer cycles using single cleavage embryos in Australia found that the live birth rate (LBR) of women receiving freeze-thawed embryos was significantly lower than those receiving fresh embryos, which may be due to embryo damage related to the freeze-thaw process [46], though HDP data were not included.

A retrospective cohort study of 560 singleton pregnancies found that the fetal birth weight in the IVF/ICSI group and the artificial insemination group was lower than that in the FET group, and this difference was already present when the estimated fetal weight was evaluated in the second trimester of pregnancy (21-23 weeks of pregnancy). The difference of fetal growth dynamics is considered to be due to the influence of the different manners of assisted reproductive technology on the invasiveness of trophoblast [47]. However, as no satisfactory model exists for studying extravillous trophoblasts and the controversial use of trophoblast cell lines, the mechanism of controlling trophoblast invasion and causing placental defects has yet to be fully understood [48]. In this study, the HRT-FET group with CL deletion was excluded, and only the fresh ET cycle and NC-FET were included for comparison (Table 5). In addition, considering that multiple births may affect the incidence of HDP, this study analyzed singleton pregnancies that were delivered alive (Tables 6 and 7). Accordingly, no difference was found in the incidence of HDP, suggesting that trophoblast damage caused by cryopreservation may not affect the occurrence of HDP. Even when excluding the impact of multiple births on the incidence of HDP, no statistical difference was observed between EM, embryo transfer methods (fresh ET and FET), gender of live birth and incidence of HDP. However, these findings require additional in-depth research for verification.

The main limitation of this study is its retrospective study design. All patients who were not delivered in our hospital were followed up by telephone. Some telephone follow-up patients could not accurately tell classification of HDP. Because the medical records of different hospitals can not be common to each other, there are still some difficulties in the detailed classification of HDP. Due to the complexity of HDP stratification and the lack of clear HDP classification in some patients, we did not analyze HDP stratification.

## Conclusions

In conclusion, EM does not seem to affect the occurrence of HDP in ART. During the total-FET cycle, whether the formation of CL plays a protective role in the occurrence and development of HDP was evaluated. The freeze-thaw process related embryo potential damage caused by cryopreservation has no effect on the occurrence of HDP. However, as the occurrence of HDP in EM is still inconclusive, further studies are needed. These findings also emphasize the potential risk of HDP in patients with HRT-FET cycle during pregnancy follow-up. Therefore, such patients should pay more attention to the occurrence of HDP in order to reduce adverse pregnancy outcomes related to assisted reproductive technology treatment.

## Data Availability

The analysed data for the current study will be available from the corresponding author.

## Abbreviations

FET: Frozen-thawed embryo transfer
NC-FET: Frozen-thawed embryo transfer with natural cycle
HRT-FET: Frozen-thawed embryo transfer with hormone therapy
